# 3-Benzyl­sulfanyl-5-(4-phenyl-1*H*-1,2,3-triazol-1-ylmeth­yl)-4*H*-1,2,4-triazol-4-amine

**DOI:** 10.1107/S1600536808023180

**Published:** 2008-07-31

**Authors:** Qing-Zhu Chu, Huan-Ran Zhou, Xiao-Ru Zhang

**Affiliations:** a College of Chemistry and Molecular Engineering, Qingdao University of Science and Technology, 266042 Qingdao, Shandong, People’s Republic of China

## Abstract

The mol­ecule of the title compound, C_18_H_17_N_7_S, is non-planar, with a dihedral angle of 71.4 (4)° between the two triazole rings, and an angle of 15.5 (3)° between the two phenyl rings. An intra­molecular N—H⋯S hydrogen bond forms a five-membered ring.

## Related literature

For related literature, see: Allen *et al.* (1987 ▶); Barchiesi *et al.* (2000 ▶); Colanceska-Ragenovic *et al.* (2001 ▶); Kaplancıklı *et al.* (2008 ▶); Khanum *et al.* (2005 ▶); Rodriguez-Fernandez *et al.* (2005 ▶); Zhang *et al.* (2008 ▶).
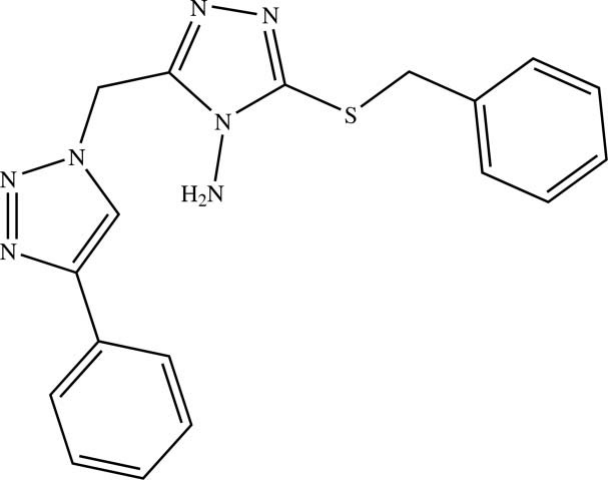


## Experimental

### 

#### Crystal data

C_18_H_17_N_7_S
*M* *_r_* = 363.45
Orthorhombic, 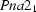

*a* = 8.0487 (15) Å
*b* = 5.4689 (10) Å
*c* = 38.721 (7) Å
*V* = 1704.4 (5) Å^3^
*Z* = 4
Mo *K*α radiation
μ = 0.21 mm^−1^
*T* = 186.5 (2) K
0.35 × 0.25 × 0.04 mm

#### Data collection

Siemens SMART 1000 CCD area-detector diffractometer
Absorption correction: multi-scan (*SADABS*; Sheldrick, 1996 ▶) *T*_min_ = 0.931, *T*_max_ = 0.992
7815 measured reflections
2854 independent reflections
2592 reflections with *I* > 2σ(*I*)
*R*_int_ = 0.052

#### Refinement

*R*[*F* ^2^ > 2σ(*F* ^2^)] = 0.078
*wR*(*F* ^2^) = 0.183
*S* = 1.20
2854 reflections
235 parameters
1 restraint
H-atom parameters constrained
Δρ_max_ = 0.48 e Å^−3^
Δρ_min_ = −0.39 e Å^−3^
Absolute structure: Flack (1983 ▶), with 2854 Freidel pairs
Flack parameter: 0.05 (19)

### 

Data collection: *SMART* (Siemens, 1996 ▶); cell refinement: *SAINT* (Siemens, 1996 ▶); data reduction: *SAINT*; program(s) used to solve structure: *SHELXS97* (Sheldrick, 2008 ▶); program(s) used to refine structure: *SHELXL97* (Sheldrick, 2008 ▶); molecular graphics: *SHELXTL* (Sheldrick, 2008 ▶); software used to prepare material for publication: *SHELXTL*, *PARST* (Nardelli, 1995 ▶) and *PLATON* (Spek, 2003 ▶).

## Supplementary Material

Crystal structure: contains datablocks global, I. DOI: 10.1107/S1600536808023180/at2597sup1.cif

Structure factors: contains datablocks I. DOI: 10.1107/S1600536808023180/at2597Isup2.hkl

Additional supplementary materials:  crystallographic information; 3D view; checkCIF report

## Figures and Tables

**Table 1:** Hydrogen-bond geometry (Å, °)

*D*—H⋯*A*	*D*—H	H⋯*A*	*D*⋯*A*	*D*—H⋯*A*
N7—H7*B*⋯S1	0.86	2.77	3.116 (6)	105

**Fig. 1.**
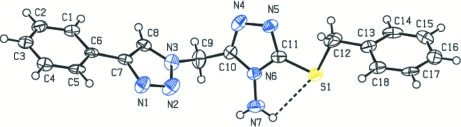
The structure of the compound (I) showing 50% probability displacement ellipsoids and the atom numbering scheme.

**Fig. 2.**
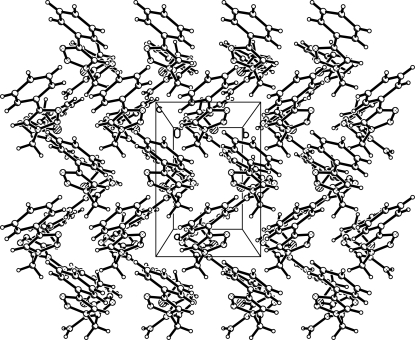
A packing diagram of (I), viewed down the c axis.

## supplementary crystallographic information

### Comment

Triazole derivatives exhibit higher antifungal activity against filamentous fungi and yeasts (Barchiesi *et al.*, 2000), and their antibacterial properties have been reported as well (Colanceska-Ragenovic *et al.*, 2001). The biological activities of various 1,2,3-triazole and 1,2,4-triazole derivatives have been extensively studied (Rodriguez-Fernandez *et al.*, 2005; Kaplancıklı *et al.*, 2008; Khanum *et al.*, 2005). As an extension of the work on structure characterization of triazole derivatives, the title compound (I) was synthesized and its structure was characterized by X-ray crystallographic analysis.

In (I) (Fig. 1), all the bond lengths and angles are within normal ranges (Allen *et al.*, 1987). A (N1–N3/C7/C8) triazole ring makes dihedral angles of 11.6 (3) and 25.6 (3)° with B (C1–C6) and C (C13–C18) phenyl rings, respectively, and of 71.4 (4)° with D (N4–N6/C10/C11) triazole ring. In addition, the dihedral angles between B and C, C and D, and B and D are 15.5 (3), 67.7 (3) and 73.7 (3)°, respectively. There exists one intramolecular hydrogen bond, N7—H7B···S1 (Table 1), forming a five-membered ring.

### Experimental

3-{[4-(4-phenyl)-1*H*-1,2,3-triazol-1-yl]methyl}-4-amino-5-thiol-1,2,4-triazole (Zhang *et al.*, 2008) (1.00 g, 3.66 mmol) was added to a mixture of 50% NaOH (*w*/*w*) 5 ml, water (30 ml) and benzyl bromide (0.87 ml, 7.32 mmol). The mixture was stirred for 5 h. The resulting precipitate was collected by filtration, washed by water and recrystallized from acetone to give compound I (1.09 g, 82.0%). Colourless single crystals suitable for an X-ray diffraction study were obtained by slow evaporation of an ethyl acetate solution.

### Refinement

All H atoms were located in difference Fourier maps and constrained to ride on their parent atoms, with C—H distances in the range 0.93–0.97 Å and N—H distances of 0.86 Å, and with *U*_iso_(H) = 1.2 *U*_eq_(C) H atoms.

### Crystal data

**Table:**

C_18_H_17_N_7_S	*F*_000_ = 760
*M**_r_* = 363.45	*D*_x_ = 1.416 Mg m^−^^3^
Orthorhombic, *P**n**a*2_1_	Mo *K*α radiation λ = 0.71073 Å
Hall symbol: P 2c -2n	Cell parameters from 1817 reflections
*a* = 8.0487 (15) Å	θ = 3.2–25.0º
*b* = 5.4689 (10) Å	µ = 0.21 mm^−^^1^
*c* = 38.721 (7) Å	*T* = 186.5 (2) K
*V* = 1704.4 (5) Å^3^	Plate, colourless
*Z* = 4	0.35 × 0.25 × 0.04 mm

### Data collection

**Table:**

Siemens SMART 1000 CCD area-detector diffractometer	2854 independent reflections
Radiation source: fine-focus sealed tube	2592 reflections with *I* > 2σ(*I*)
Monochromator: graphite	*R*_int_ = 0.052
Detector resolution: 8.33 pixels mm^-1^	θ_max_ = 25.0º
*T* = 293(2) K	θ_min_ = 2.1º
ω scans	*h* = −9→9
Absorption correction: multi-scan(SADABS; Sheldrick, 1996)	*k* = −6→5
*T*_min_ = 0.931, *T*_max_ = 0.992	*l* = −40→46
7815 measured reflections	

### Refinement

**Table:**

Refinement on *F*^2^	Hydrogen site location: inferred from neighbouring sites
Least-squares matrix: full	H-atom parameters constrained
*R*[*F*^2^ > 2σ(*F*^2^)] = 0.078	*w* = 1/[σ^2^(*F*_o_^2^) + (0.0579*P*)^2^ + 3.955*P*] where *P* = (*F*_o_^2^ + 2*F*_c_^2^)/3
*wR*(*F*^2^) = 0.183	(Δ/σ)_max_ = 0.002
*S* = 1.20	Δρ_max_ = 0.48 e Å^−^^3^
2854 reflections	Δρ_min_ = −0.39 e Å^−^^3^
235 parameters	Extinction correction: none
1 restraint	Absolute structure: Flack (1983), with 2854 Freidel pairs
Primary atom site location: structure-invariant direct methods	Flack parameter: 0.05 (19)
Secondary atom site location: difference Fourier map	

### Special details

**Table:**

Geometry. All e.s.d.'s (except the e.s.d. in the dihedral angle between two l.s. planes) are estimated using the full covariance matrix. The cell e.s.d.'s are taken into account individually in the estimation of e.s.d.'s in distances, angles and torsion angles; correlations between e.s.d.'s in cell parameters are only used when they are defined by crystal symmetry. An approximate (isotropic) treatment of cell e.s.d.'s is used for estimating e.s.d.'s involving l.s. planes.
Refinement. Refinement of *F*^2^ against ALL reflections. The weighted *R*-factor *wR* and goodness of fit *S* are based on *F*^2^, conventional *R*-factors *R* are based on *F*, with *F* set to zero for negative *F*^2^. The threshold expression of *F*^2^ > σ(*F*^2^) is used only for calculating *R*-factors(gt) *etc*. and is not relevant to the choice of reflections for refinement. *R*-factors based on *F*^2^ are statistically about twice as large as those based on *F*, and *R*- factors based on ALL data will be even larger.

### Fractional atomic coordinates and isotropic or equivalent isotropic displacement parameters (Å^2^)

**Table:**

	*x*	*y*	*z*	*U*_iso_*/*U*_eq_	
S1	0.0735 (2)	0.5751 (3)	0.34992 (5)	0.0348 (4)	
N5	−0.0030 (7)	0.2276 (10)	0.39829 (15)	0.0349 (13)	
C11	0.0723 (7)	0.4329 (11)	0.38974 (17)	0.0284 (14)	
N6	0.1621 (6)	0.5157 (9)	0.41739 (14)	0.0299 (12)	
C1	−0.0955 (7)	0.3110 (10)	0.60677 (16)	0.0266 (14)	
H1A	−0.0314	0.1797	0.5993	0.032*	
C7	−0.0485 (7)	0.5171 (11)	0.55000 (16)	0.0245 (14)	
N4	0.0366 (7)	0.1806 (11)	0.43251 (16)	0.0398 (15)	
C13	−0.0838 (8)	0.4412 (12)	0.29074 (17)	0.0344 (16)	
N1	−0.0513 (8)	0.7223 (11)	0.53032 (15)	0.0428 (15)	
C3	−0.2600 (7)	0.4969 (12)	0.65095 (18)	0.0305 (14)	
H3A	−0.3061	0.4923	0.6730	0.037*	
N2	0.0363 (8)	0.6895 (11)	0.50221 (17)	0.0464 (16)	
C5	−0.2313 (8)	0.6963 (10)	0.59606 (18)	0.0297 (15)	
H5B	−0.2576	0.8261	0.5815	0.036*	
C14	−0.0064 (8)	0.2866 (11)	0.26757 (19)	0.0356 (17)	
H14A	0.0496	0.1498	0.2758	0.043*	
N3	0.0979 (6)	0.4597 (10)	0.50423 (14)	0.0322 (13)	
C16	−0.0919 (9)	0.5334 (14)	0.2199 (2)	0.0419 (18)	
H16A	−0.0967	0.5634	0.1963	0.050*	
N7	0.2650 (7)	0.7185 (10)	0.41686 (15)	0.0423 (15)	
H7A	0.3227	0.7555	0.4348	0.051*	
H7B	0.2712	0.8075	0.3986	0.051*	
C2	−0.1574 (9)	0.3117 (11)	0.63972 (17)	0.0354 (16)	
H2B	−0.1299	0.1853	0.6547	0.042*	
C4	−0.2935 (8)	0.6914 (11)	0.62878 (17)	0.0302 (15)	
H4B	−0.3594	0.8201	0.6365	0.036*	
C6	−0.1276 (7)	0.5067 (11)	0.58396 (16)	0.0258 (14)	
C17	−0.1648 (8)	0.6881 (11)	0.24299 (18)	0.0334 (16)	
H17A	−0.2206	0.8245	0.2346	0.040*	
C10	0.1372 (7)	0.3584 (12)	0.44322 (16)	0.0286 (14)	
C18	−0.1598 (8)	0.6523 (11)	0.27809 (18)	0.0321 (15)	
H18A	−0.2062	0.7662	0.2931	0.038*	
C8	0.0472 (7)	0.3493 (11)	0.53312 (16)	0.0271 (14)	
H8A	0.0720	0.1910	0.5403	0.033*	
C15	−0.0103 (8)	0.3297 (13)	0.2329 (2)	0.0386 (17)	
H15A	0.0421	0.2220	0.2178	0.046*	
C9	0.2124 (8)	0.3702 (15)	0.47810 (17)	0.0405 (17)	
H9A	0.2502	0.2082	0.4846	0.049*	
H9B	0.3088	0.4766	0.4774	0.049*	
C12	−0.0816 (10)	0.3877 (15)	0.3288 (2)	0.053 (2)	
H12A	−0.0565	0.2162	0.3325	0.063*	
H12B	−0.1901	0.4213	0.3386	0.063*	

### Atomic displacement parameters (Å^2^)

**Table:**

	*U*^11^	*U*^22^	*U*^33^	*U*^12^	*U*^13^	*U*^23^
S1	0.0327 (8)	0.0356 (9)	0.0361 (9)	−0.0077 (7)	−0.0003 (8)	0.0057 (9)
N5	0.029 (3)	0.035 (3)	0.041 (3)	−0.009 (3)	0.000 (2)	0.007 (3)
C11	0.019 (3)	0.024 (3)	0.043 (4)	0.001 (3)	0.005 (3)	−0.001 (3)
N6	0.027 (3)	0.026 (3)	0.037 (3)	0.001 (2)	0.002 (2)	0.001 (3)
C1	0.018 (3)	0.019 (3)	0.042 (4)	0.004 (3)	−0.004 (3)	0.003 (3)
C7	0.019 (3)	0.017 (3)	0.037 (4)	0.003 (2)	−0.005 (2)	−0.002 (3)
N4	0.033 (3)	0.043 (4)	0.043 (4)	0.004 (3)	0.009 (3)	0.014 (3)
C13	0.032 (4)	0.029 (3)	0.042 (4)	−0.006 (3)	−0.006 (3)	0.008 (3)
N1	0.046 (3)	0.039 (3)	0.043 (4)	0.014 (3)	0.011 (3)	0.011 (3)
C3	0.020 (3)	0.032 (4)	0.040 (4)	−0.006 (3)	0.000 (3)	−0.001 (3)
N2	0.047 (4)	0.037 (4)	0.055 (4)	0.018 (3)	0.011 (3)	0.016 (3)
C5	0.032 (3)	0.010 (3)	0.048 (4)	0.004 (3)	−0.007 (3)	0.002 (2)
C14	0.021 (3)	0.015 (3)	0.071 (5)	0.007 (3)	−0.011 (3)	0.006 (3)
N3	0.016 (2)	0.041 (3)	0.039 (3)	0.011 (2)	0.000 (2)	0.002 (3)
C16	0.036 (4)	0.051 (5)	0.039 (4)	−0.019 (4)	−0.004 (3)	−0.001 (4)
N7	0.042 (3)	0.033 (3)	0.051 (4)	−0.011 (3)	−0.008 (3)	0.006 (3)
C2	0.042 (4)	0.025 (3)	0.040 (4)	−0.005 (3)	−0.002 (3)	0.005 (3)
C4	0.024 (3)	0.023 (3)	0.044 (4)	0.011 (3)	0.001 (3)	−0.004 (3)
C6	0.025 (3)	0.020 (3)	0.032 (4)	−0.008 (3)	−0.007 (3)	0.001 (2)
C17	0.026 (3)	0.018 (3)	0.056 (5)	−0.009 (3)	0.005 (3)	0.001 (3)
C10	0.024 (3)	0.030 (3)	0.031 (4)	0.005 (3)	0.003 (3)	0.004 (3)
C18	0.023 (3)	0.023 (3)	0.050 (4)	0.004 (3)	0.006 (3)	0.000 (3)
C8	0.029 (3)	0.015 (3)	0.037 (4)	0.011 (3)	−0.005 (3)	−0.001 (3)
C15	0.030 (4)	0.034 (4)	0.052 (5)	−0.001 (3)	−0.001 (3)	−0.015 (3)
C9	0.026 (3)	0.060 (5)	0.035 (4)	0.007 (3)	0.007 (3)	−0.001 (4)
C12	0.045 (4)	0.060 (5)	0.053 (5)	−0.034 (4)	−0.008 (4)	0.012 (4)

### Geometric parameters (Å, °)

**Table:**

S1—C11	1.727 (6)	C5—C6	1.411 (9)
S1—C12	1.810 (7)	C5—H5B	0.9300
N5—C11	1.318 (8)	C14—C15	1.365 (10)
N5—N4	1.387 (8)	C14—H14A	0.9300
C11—N6	1.369 (8)	N3—C8	1.335 (8)
N6—C10	1.334 (8)	N3—C9	1.454 (8)
N6—N7	1.385 (7)	C16—C17	1.363 (10)
C1—C2	1.370 (9)	C16—C15	1.386 (10)
C1—C6	1.412 (8)	C16—H16A	0.9300
C1—H1A	0.9300	N7—H7A	0.8600
C7—N1	1.357 (8)	N7—H7B	0.8600
C7—C8	1.365 (8)	C2—H2B	0.9300
C7—C6	1.462 (8)	C4—H4B	0.9300
N4—C10	1.332 (9)	C17—C18	1.374 (9)
C13—C14	1.381 (10)	C17—H17A	0.9300
C13—C18	1.395 (9)	C10—C9	1.481 (9)
C13—C12	1.503 (10)	C18—H18A	0.9300
N1—N2	1.309 (8)	C8—H8A	0.9300
C3—C2	1.377 (9)	C15—H15A	0.9300
C3—C4	1.393 (9)	C9—H9A	0.9700
C3—H3A	0.9300	C9—H9B	0.9700
N2—N3	1.353 (8)	C12—H12A	0.9700
C5—C4	1.362 (9)	C12—H12B	0.9700
			
C11—S1—C12	98.3 (3)	H7A—N7—H7B	120.0
C11—N5—N4	107.0 (5)	C1—C2—C3	121.0 (6)
N5—C11—N6	109.2 (5)	C1—C2—H2B	119.5
N5—C11—S1	127.6 (5)	C3—C2—H2B	119.5
N6—C11—S1	123.1 (4)	C5—C4—C3	121.2 (6)
C10—N6—C11	107.1 (5)	C5—C4—H4B	119.4
C10—N6—N7	128.1 (6)	C3—C4—H4B	119.4
C11—N6—N7	124.7 (5)	C5—C6—C1	117.2 (6)
C2—C1—C6	120.9 (6)	C5—C6—C7	121.9 (6)
C2—C1—H1A	119.5	C1—C6—C7	120.8 (6)
C6—C1—H1A	119.5	C16—C17—C18	123.2 (7)
N1—C7—C8	107.3 (6)	C16—C17—H17A	118.4
N1—C7—C6	122.0 (5)	C18—C17—H17A	118.4
C8—C7—C6	130.6 (6)	N4—C10—N6	109.2 (6)
C10—N4—N5	107.6 (5)	N4—C10—C9	124.3 (6)
C14—C13—C18	118.4 (6)	N6—C10—C9	126.5 (6)
C14—C13—C12	120.8 (6)	C17—C18—C13	118.6 (6)
C18—C13—C12	120.7 (7)	C17—C18—H18A	120.7
N2—N1—C7	110.1 (5)	C13—C18—H18A	120.7
C2—C3—C4	118.9 (6)	N3—C8—C7	105.6 (5)
C2—C3—H3A	120.6	N3—C8—H8A	127.2
C4—C3—H3A	120.6	C7—C8—H8A	127.2
N1—N2—N3	106.0 (5)	C14—C15—C16	120.3 (7)
C4—C5—C6	120.7 (6)	C14—C15—H15A	119.8
C4—C5—H5B	119.6	C16—C15—H15A	119.8
C6—C5—H5B	119.6	N3—C9—C10	113.0 (5)
C15—C14—C13	121.6 (6)	N3—C9—H9A	109.0
C15—C14—H14A	119.2	C10—C9—H9A	109.0
C13—C14—H14A	119.2	N3—C9—H9B	109.0
C8—N3—N2	110.9 (5)	C10—C9—H9B	109.0
C8—N3—C9	128.7 (5)	H9A—C9—H9B	107.8
N2—N3—C9	120.3 (6)	C13—C12—S1	109.9 (5)
C17—C16—C15	117.8 (7)	C13—C12—H12A	109.7
C17—C16—H16A	121.1	S1—C12—H12A	109.7
C15—C16—H16A	121.1	C13—C12—H12B	109.7
N6—N7—H7A	120.0	S1—C12—H12B	109.7
N6—N7—H7B	120.0	H12A—C12—H12B	108.2
			
N4—N5—C11—N6	1.3 (7)	N1—C7—C6—C1	−167.2 (6)
N4—N5—C11—S1	177.6 (5)	C8—C7—C6—C1	7.4 (10)
C12—S1—C11—N5	9.0 (7)	C15—C16—C17—C18	0.5 (10)
C12—S1—C11—N6	−175.1 (6)	N5—N4—C10—N6	0.3 (7)
N5—C11—N6—C10	−1.1 (7)	N5—N4—C10—C9	−177.8 (6)
S1—C11—N6—C10	−177.7 (5)	C11—N6—C10—N4	0.5 (7)
N5—C11—N6—N7	175.7 (6)	N7—N6—C10—N4	−176.2 (6)
S1—C11—N6—N7	−0.8 (8)	C11—N6—C10—C9	178.5 (6)
C11—N5—N4—C10	−1.0 (7)	N7—N6—C10—C9	1.8 (10)
C8—C7—N1—N2	0.4 (8)	C16—C17—C18—C13	−3.3 (10)
C6—C7—N1—N2	176.2 (6)	C14—C13—C18—C17	4.6 (9)
C7—N1—N2—N3	−0.7 (8)	C12—C13—C18—C17	−177.3 (6)
C18—C13—C14—C15	−3.3 (10)	N2—N3—C8—C7	−0.6 (7)
C12—C13—C14—C15	178.6 (6)	C9—N3—C8—C7	175.1 (6)
N1—N2—N3—C8	0.8 (7)	N1—C7—C8—N3	0.1 (7)
N1—N2—N3—C9	−175.3 (6)	C6—C7—C8—N3	−175.1 (6)
C6—C1—C2—C3	3.4 (9)	C13—C14—C15—C16	0.5 (10)
C4—C3—C2—C1	−3.2 (9)	C17—C16—C15—C14	0.9 (10)
C6—C5—C4—C3	−1.6 (10)	C8—N3—C9—C10	121.5 (7)
C2—C3—C4—C5	2.3 (10)	N2—N3—C9—C10	−63.1 (8)
C4—C5—C6—C1	1.7 (9)	N4—C10—C9—N3	−79.2 (8)
C4—C5—C6—C7	−175.0 (6)	N6—C10—C9—N3	103.1 (8)
C2—C1—C6—C5	−2.6 (8)	C14—C13—C12—S1	101.0 (7)
C2—C1—C6—C7	174.1 (6)	C18—C13—C12—S1	−77.1 (8)
N1—C7—C6—C5	9.3 (9)	C11—S1—C12—C13	−169.3 (6)
C8—C7—C6—C5	−176.0 (6)		

### Hydrogen-bond geometry (Å, °)

**Table:**

*D*—H···*A*	*D*—H	H···*A*	*D*···*A*	*D*—H···*A*
N7—H7B···S1	0.86	2.78	3.116 (6)	105
